# Bridging Development and Disruption: Comprehensive Insights into Focal Cortical Dysplasia and Epileptic Management

**DOI:** 10.7759/cureus.45996

**Published:** 2023-09-26

**Authors:** Syed Naveed Mohsin, Han Grezenko, Saadia Khan, Filagot D Eshete, Shraddha Shrestha, Muhammad Kamran, Maryam Affaf, Ayat Jama, Rayan W Gasim, Dewan Zubaer Ahmad, Indresh Yadav, Sidra Arif, Anil K. C., Abdul Saboor Khaliq

**Affiliations:** 1 General Surgery, Cavan General Hospital, Cavan, IRL; 2 Translational Neuroscience, Barrow Neurological Institute, Phoenix, USA; 3 Community Medicine, Khyber Girls Medical College, Peshawar, PAK; 4 General Surgery, Jimma University, Jimma, ETH; 5 Internal Medicine, Nepal Korea Friendship Municipality Hospital, Bhaktapur, NPL; 6 Internal Medicine, Mayo Hospital, Lahore, PAK; 7 Internal Medicine, Women’s Medical and Dental College, Abbottabad, PAK; 8 Internal Medicine, Nishtar Medical University, Multan, PAK; 9 Internal Medicine, University of Khartoum, Khartoum, SDN; 10 Medicine, University of Dhaka, Dhaka, BGD; 11 Internal Medicine, Samar Hospital and Research Center Pvt. Ltd., Janakpur, NPL; 12 Internal Medicine, Community Based Medical College, Mymensingh, BGD; 13 Urology, Jinnah Postgraduate Medical Center, Karachi, PAK; 14 Medicine and Surgery, Patan Academy of Health Sciences, Kathmandu, NPL; 15 Internal Medicine and Neurology, California Institute of Behavioral Neurosciences & Psychology, California, USA; 16 Internal Medicine, Allama Iqbal Medical College, Lahore, PAK

**Keywords:** epilepsy, cns complications, neurology and critical care, future management, focal cortical dysplasia

## Abstract

Focal cortical dysplasia (FCD) is a prominent neurological disorder characterized by disruptions in localized brain cell organization and development. This narrative review delineates the multi-faceted nature of FCD, emphasizing its correlation with drug-resistant epilepsy, predominantly in children and young adults. We explore the historical context of FCD, highlighting its indispensable role in shaping our comprehension of epilepsy and cortical anomalies. The clinical spectrum of FCD is broad, encompassing diverse seizure patterns, cognitive impairments, and associated neuropsychiatric disorders. We underscore the importance of differential diagnosis, with techniques ranging from electroencephalogram (EEG) interpretations to microscopic evaluations, and discuss advanced diagnostic modalities, such as the 3T magnetic resonance imaging (MRI) epilepsy protocols. Therapeutically, while anti-seizure medications are often first-line interventions, surgically refractory cases necessitate more invasive procedures, underscoring the importance of individualized treatment. Furthermore, the review touches upon the prognostic aspects of FCD, highlighting the importance of personalized care regimens, and provides insights into emerging therapeutic avenues, including the potential of the mammalian target of rapamycin (mTOR) pathway. Conclusively, this review accentuates the complex relationship between brain development and epileptogenicity inherent to FCD and underscores the promise of future research in enhancing patient outcomes.

## Introduction and background

Epilepsy, a prevalent and chronic neurological disorder, emerges from aberrant brain electrical activities driven by an imbalance between excitatory and inhibitory neuronal impulses [1]. This condition, affecting an estimated 50 million people globally, doesn’t just culminate in seizures. Patients often confront societal stigmas, misconceptions, and resultant prejudices, leading to mental distress, societal exclusion, and curtailed opportunities in education, employment, and broader societal activities [2].

The intricate web of epilepsy's etiology is woven from diverse strands. These include structural anomalies like traumatic brain injuries and tumors [3], genetic factors including inherited disorders and familial histories of epilepsy, and environmental triggers such as substance abuse and infectious brain diseases like meningitis [4]. Notably, events like high fevers, especially in children, can exacerbate the risk of developing epilepsy [5].

In the US, in 2015, reported that approximately 3.4 million of its residents, cutting across all ages, were living with active epilepsy, which is about 1.2% of its population [6]. Such statistics elucidate that epilepsy's ramifications permeate beyond patients, affecting families, caregivers, and broader communities. Epilepsy’s onset can be at any life stage, with triggers and causes oscillating based on age groups.

Among the myriad structural anomalies associated with epilepsy, focal cortical dysplasia (FCD) looms large. Defined as a localized aberration in the natural progression and organization of brain cells, especially neurons [7], FCD is intrinsic and congenital. It's closely linked to the advent of medically refractory epilepsy and has been a predominant factor in epilepsy cases across pediatric and adult populations. This connection between FCD and epilepsy was first elucidated in 1971, when David Taylor and his team detailed its features in 10 patients with drug-resistant epilepsy [8]. This groundbreaking work earmarked FCD as a unique cortical developmental disorder, offering invaluable insights into epilepsy's mechanisms.

While focal cortical dysplasia (FCD) has been established as a vital factor in epilepsy cases, its diverse manifestations and impact on patients warrant a closer examination. FCD is not a uniform entity; instead, it encompasses a spectrum of structural brain abnormalities, each with its distinct characteristics. These variations in presentation make the diagnosis and treatment of FCD a complex endeavor. Moreover, recent advancements in neuroimaging techniques, such as high-resolution magnetic resonance imaging (MRI) and positron emission tomography (PET) scans, have improved our ability to detect and classify different FCD subtypes. Understanding these nuances is essential for tailoring precise therapeutic strategies and enhancing patient outcomes. As we delve into the intricacies of FCD, we uncover a dynamic landscape that challenges our perceptions of cortical development and its implications for epilepsy.

## Review

Overview of FCD

FCD stands as a distinct disorder characterized by localized disruptions in the typical architecture and evolution of brain cells, with neurons being the primary cells affected [7]. This developmental aberration is inherent and frequently serves as a precursor to the onset of epilepsy, especially those forms resistant to conventional therapeutic interventions [8]. FCD is a major causative factor behind epilepsy in diverse age groups, from children to adults. Its anomalous cellular development patterns interfere with the brain's electrical dynamics, leading to seizures and other neurological manifestations [9]. Ever since David Taylor and his peers cast a spotlight on FCD's distinct attributes in their 1971 study involving patients with drug-resistant epilepsy [10], it has been spotlighted as a critical factor behind medically refractory epilepsy. The insights gleaned from studying FCD over the years have enriched our grasp on epilepsy, enabling us to devise more tailored diagnostic and therapeutic approaches [11].

It's important to distinguish FCD from other types of focal or generalized epilepsy. Unlike generalized epilepsy, where seizures can originate from multiple areas in the brain, FCD is defined by its localized disruptions in cortical architecture. Furthermore, while other forms of focal epilepsy may arise from lesions such as tumors or scars, FCD is characterized by developmental abnormalities that are intrinsic to the brain tissue itself. This makes FCD a particularly challenging form of epilepsy to manage, often requiring specialized diagnostic and therapeutic approaches. In addition, FCD often manifests as drug-resistant epilepsy, setting it apart from other epilepsy types that may respond more favorably to conventional anti-seizure medications. This unique profile of FCD has necessitated the development of specialized diagnostic procedures and tailored treatments, further emphasizing its distinct position within the broader spectrum of epileptic disorders.

Pathophysiology of FCD

Origin and Development

The pathophysiology of FCD is intricately tied to deviations in the processes of neuronal proliferation, differentiation, and migration during the cortical development phase of embryogenesis. These anomalies result in disordered cortical lamination, presenting with architectural deviations that can range from a subtle blurring of the gray-white boundary to pronounced multilobar lesions filled with dysmorphic neurons and balloon cells penetrating deep into the white matter [12]. This broad spectrum of manifestations arises from interruptions at various junctures of corticogenesis, attributable to a mix of genetic, molecular, and environmental influences [13]. Typically, the embryonic window for such cortical malformations spans from the proliferation phase of neuronal precursor cells around weeks 12-24, extending to the migration and subsequent organization of layer-specific neurons from weeks 24 to 34.

Neuronal Mechanisms Leading to Seizures

FCD can be a precursor to seizures, notably focal-onset seizures that might progress to generalized tonic-clonic activity. The initial manifestations of these seizures can offer insights into the specific brain region affected. The genesis of seizures in this context can be attributed to dysmorphic neurons, aberrant connectivity patterns, and regions with pronounced epileptogenic potential [8]. The underpinning cause for seizures associated with FCD is disruptions in neuronal connectivity, propelled by genetic modifications that engender hyperexcitability and synchronization within neural networks [14].

Types of FCD

**Figure 1:**
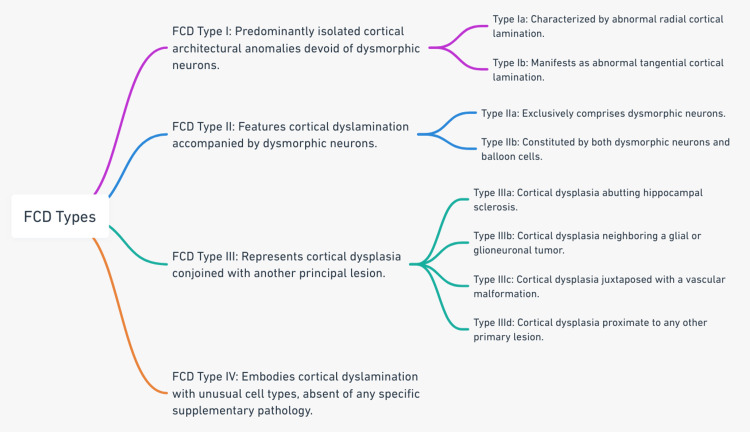
Classification of types of FCD. FCD: focal cortical dysplasia. Note: The image is created by the author.

The landscape of FCD classification has witnessed multiple proposals, but the 2011 International League Against Epilepsy (ILAE) consensus classification stands out as the predominant system [15]. The classification is given in the diagram (Figure 1).

Correctly categorizing the subtype holds significant clinical relevance, as it correlates with imaging traits, epileptogenic propensity, and post-surgical outcomes. However, it's worth noting that there exists heterogeneity within these subtypes, and not every instance aligns perfectly with this classification. As such, contemporary research endeavors to further refine FCD subclassifications, leveraging imaging techniques, genetic insights, and neuropathological evaluations [15].

Clinical presentation

Symptom Onset and Progression

The clinical presentation of FCD displays considerable variability, which can be attributed to the heterogeneity of the underlying cortical lesions and disturbances in neural networks. Seizures are the most common initial manifestation, but FCD might also be identified incidentally or in conjunction with developmental delays or behavioral anomalies [16]. The comprehensive range of seizure semiology patterns has been addressed in previous sections.

Besides seizures, a range of cognitive and psychiatric comorbidities frequently accompany FCD. Retrospective studies indicate that cognitive impairments are prevalent in nearly 50% of surgical FCD cases, encompassing deficits such as reduced IQ, diminished verbal and visual memory, decreased processing speed, executive dysfunction, and inattention [17,18]. Intellectual disabilities can affect as many as 25% of patients, while psychiatric disorders such as autism spectrum disorder, attention-deficit/hyperactivity disorder (ADHD), anxiety, depression, and psychotic symptoms are evident in 5-30% of cases [19]. Observational data places the prevalence of neuropsychiatric co-morbidity in FCD between 37 and 62% [20].

While seizures are a common manifestation across various types of epilepsy, the seizure patterns in FCD often present unique challenges for diagnosis and management. The seizures associated with FCD are frequently resistant to standard anti-seizure medications, which is not always the case in other forms of epilepsy. Moreover, the localized nature of cortical disruptions in FCD can give rise to highly specialized seizure patterns that may be focal to a particular region of the brain, further complicating the diagnosis.

In addition to seizures, FCD is uniquely characterized by a higher prevalence of specific cognitive and neuropsychiatric comorbidities, such as autism spectrum disorder and ADHD. These distinct features underscore the need for a tailored approach in both diagnosis and treatment, emphasizing FCD's unique clinical landscape.

Associated Neurological Findings

On physical examination, focal motor deficits such as hemiparesis or hemidystonia that correspond to the sites of cortical injury are identifiable in 15-45% of individuals diagnosed with FCD. Even though more subtle neurological signs might manifest, a significant percentage, up to 70% of patients with FCD confirmed via imaging, may exhibit a completely normal physical examination [16]. Additionally, non-specific symptoms like global developmental delays are prevalent, particularly among pediatric FCD populations. The overlap with the manifestations of other focal epilepsy etiologies underscores the critical importance of detailed patient history, thorough clinical assessment, and the utilization of multimodal confirmatory tests.

Differential Diagnosis

Differentiating FCD from other epileptogenic causes is paramount, especially since drug-resistant epilepsy often stems from FCD, particularly in children and young adults. Key diagnostic considerations include:

Electroencephalogram (EEG): While EEG findings might not be exclusive to FCD and can be seen in other epilepsy cases, they can show background slowing in the region affected by FCD. Focal rapid activity detected on the EEG is more indicative of FCD [8].

Microscopic review: The definitive diagnosis of FCD is often provided through the microscopic examination of surgically excised brain tissue, guided by the categorization standards set by the International League Against Epilepsy [21].

Clinical presentation: Treating seizures associated with FCD can be challenging. Tailored strategies are essential for the effective management of epilepsy and its cognitive repercussions [22].

Genetic association: Research endeavors are underway to elucidate the implicated genes and explore potential therapeutic interventions targeting these genetic factors.

Diagnostic modalities for FCD

Imaging Modalities

Focal cortical dysplasia (FCD) often demands a comprehensive array of imaging techniques to aid in its diagnosis and management. Magnetic resonance imaging (MRI) is the frontline modality owing to its capability to delineate cortical architecture and white matter tracts. It can depict the hallmark MRI features of FCD, which include cortical thickening, blurring of the gray-white matter boundary, and abnormal white matter signal intensity [23]. Advanced MRI sequences, such as fluid-attenuated inversion recovery (FLAIR) and T2-weighted images, enhance the sensitivity of detecting subtle FCD lesions. Additionally, magnetic resonance spectroscopy (MRS) can be utilized to assess the metabolic profile of the dysplastic cortex, providing further insights into the abnormal tissue [24].

Functional imaging, such as positron emission tomography (PET) and single-photon emission computed tomography (SPECT), can help localize epileptogenic zones, especially in patients where MRI fails to demonstrate a visible lesion. These modalities measure regional cerebral blood flow and metabolism, offering insights into areas of hypometabolism, which are often congruent with epileptogenic foci [25].

Electroencephalogram (EEG)

EEG plays an indispensable role in the diagnosis and management of FCD, capturing the electrical activity of the brain and assisting in the localization of the seizure focus. In cases of FCD, the EEG may reveal focal epileptiform discharges or continuous slow-wave activity, especially during sleep, that corresponds to the location of the dysplasia [26]. In individuals where standard EEG findings are inconclusive, prolonged video-EEG monitoring or intracranial EEG might be required. These advanced techniques provide a more detailed assessment of the seizure onset zone and can guide pre-surgical evaluations [27].

These specialized diagnostic criteria underscore the unique challenges and considerations in identifying FCD, requiring a targeted approach distinct from the diagnostic strategies employed for other forms of epilepsy.

Neuropsychological Testing

The cognitive and psychiatric comorbidities associated with FCD necessitate a thorough neuropsychological evaluation. This assessment aids in identifying specific areas of cognitive strength and weakness, informing tailored intervention strategies. Neuropsychological tests can evaluate various domains, including memory, attention, executive function, language, and visuospatial abilities [28]. For patients being considered for surgical intervention, these tests not only provide a pre-surgical cognitive baseline but also help predict post-surgical cognitive outcomes. Furthermore, understanding the neuropsychological profile can assist in elucidating the functional impact of the lesion and guide rehabilitative interventions [29].

Medical management of FCD

Antiepileptic Drugs (AEDs)

The cornerstone of FCD management revolves around the use of anti-seizure medications, often heralded as the initial line of treatment. Despite the challenging prognosis for complete seizure remission, several first-line agents have emerged based on evidence and expert consensus, including valproate, levetiracetam, lamotrigine, and carbamazepine [30]. Notably, while around 30-50% of patients attain a seizure-free status on monotherapy, this figure can exhibit significant variation across different studies [31].

In situations where two first-line AEDs fail to provide adequate seizure control, clinicians might pivot towards adjunctive medications. Some of the options, backed by limited yet growing evidence, encompass topiramate, zonisamide, lacosamide, perampanel, fenfluramine, and cannabidiol [32,33]. Given the resilience of FCD to treatment, combination therapy-employing two or more drugs simultaneously-often becomes an indispensable strategy. Moreover, drugs like vigabatrin has been earmarked for infants with FCD due to its proven efficacy against infantile spasms.

Dietary Approaches

Beyond the pharmacological realm, dietary interventions have demonstrated efficacy, especially in younger populations. Additionally, the ketogenic diet has gained traction as a viable non-pharmacological alternative, showcasing promising results in pediatric FCD populations as per retrospective analyses [34].

Challenges in Medical Management

Despite the armamentarium of treatment options, a significant cohort of patients grapples with intractable seizures. This reality becomes manifest after multiple medication trials spanning one to two years fail to bring seizures under control, pushing clinicians towards considering surgical interventions. Yet, the landscape of FCD management isn't entirely bleak; the ongoing evolution and introduction of novel antiseizure agents bring forth a renewed sense of optimism, heralding the potential for enhanced therapeutic avenues in the foreseeable future [35].

These specificities in medical management underscore the necessity for a tailored approach to treating FCD, given its unique resistance patterns and the distinct efficacy of certain medications and dietary approaches.

Surgical interventions for FCD

Resection Surgery

For those patients whose epilepsy, stemming from unilateral FCD lesions, proves resistant to medications, resective surgery emerges as a pivotal curative avenue. Over the decades since the foundational studies of the 1990s, surgical methods have undergone extensive refinement [36]. The gamut of surgical choices encompasses lesionectomy, lobectomy, multi-lobar disconnections, and hemispherotomy. The selection of a specific approach is contingent on the precise location and size of the dysplastic cortex. By leveraging an array of mapping techniques, the overarching goal is to excise or functionally disengage the epileptogenic zone ensconced within one hemisphere. According to meta-analyses, this surgical modality has secured seizure freedom rates oscillating between 60 and 90%. Notably, the highest success rates have been affiliated with type I FCD and both temporal and extratemporal lesions.

Laser Interstitial Thermal Therapy (LITT)

Advancements in technology have birthed minimally invasive surgical alternatives. One such method is MRI-guided laser interstitial thermal therapy (LITT) [37]. This technique is particularly alluring when the FCD lesions are either non-resectable or scattered across multiple foci. This is in contrast to other forms of epilepsy, where LITT is less commonly applied due to the more diffuse nature of the disease. In situations where LITT might not be feasible, palliative procedures such as corpus callosotomy or multiple subpial transections can be employed, bearing the promise of a reduction in seizure frequency.

Neurostimulation Approaches

Beyond the realm of traditional surgeries lies the domain of neuromodulatory interventions. These include vagus nerve stimulation, responsive neurostimulation devices, and deep brain stimulation methodologies [38]. While these interventions might not ensure a complete cure, they can be instrumental in ameliorating the quality of life for patients. This is achieved primarily by diminishing the frequency and severity of seizures.

Neuromodulatory techniques are generally considered secondary options in FCD, owing to the higher success rates achieved with targeted surgical resections. These techniques may, however, offer benefits in cases where resective surgery is not possible due to the risk to essential cortical regions. Additionally, the high rate of medication-resistant epilepsy in FCD often accelerates the consideration for surgical interventions, making pre-surgical evaluations and postoperative monitoring crucial aspects of FCD management.

It's imperative to underscore the importance of meticulous postoperative monitoring. This vigilance is essential as, in certain instances, patients might necessitate subsequent surgeries. This could be due to reasons like an incomplete initial resection or the progression of the disease to include multiple foci. Even though recurrent seizures after surgery can be linked to a myriad of factors, such as FCD type II pathology, dual pathology, extensive lesions, incomplete MRI resection, and multifocality, repeated surgeries have demonstrated commendable success in achieving seizure freedom for a considerable segment of affected patients [39].

Prognosis and long-term management of FCD

Post-surgical Outcomes

The multifaceted challenges posed by FCD range from neuropsychiatric comorbidities to the repercussions of anti-seizure medication. Such obstacles can markedly diminish the quality of life for those affected. Interventions like cognitive rehabilitation and behavioral treatments can be instrumental in addressing learning disabilities, social interaction difficulties, emotional challenges like anxiety and depression, and the side effects of medication [40]. After surgical interventions, motor-related challenges such as hemiparesis often show remarkable improvement. Nevertheless, some residual deficits might persist, underscoring the need for continued physical and occupational therapy. When assessed in terms of seizure control, only around 33% attain complete control with medications. Contrastingly, surgical interventions boast a success rate of 60-90% in terms of postoperative seizure freedom [41]. Yet, it's crucial to note that a segment ranging from 10 to 20% witnesses a recurrence of seizures even after a year of successful surgery. In the grand scheme, life-long complete seizure remission is a reality only for one-third to two-thirds of FCD patients. Factors influencing such outcomes include the age at which the surgery was conducted and the efficacy of early seizure control, with both parameters indicating a higher probability of sustained remission [42].

Long-Term Follow-Up

The journey post-surgery can be intricate, with observational studies suggesting that up to 35% of patients might require subsequent epilepsy surgeries. The necessity for such follow-up surgeries can arise due to the emergence of new epileptogenic regions or the persistence of previous ones. Nevertheless, despite the challenges and potential risks, the overarching narrative remains positive. A significant proportion of patients, when equipped with a tailor-made management plan, witness substantial enhancements in their quality of life and overall functionality, especially if the debilitating seizures are brought under control.

Emerging therapies and future directions

In recent years, there has been a significant surge in advancements regarding the diagnosis and treatment of focal cortical dysplasia (FCD), paving the way for exciting prospects. One of the pivotal strides has been in the realm of therapeutic interventions for drug-resistant epilepsy. Especially in pediatric patients, a deeper grasp of FCD's pathophysiology has ignited the development of innovative therapies that aim to tackle the root causes of FCD-induced epilepsy rather than just alleviating its symptoms [43].

On the surgical front, FCD has emerged as a predominant factor among children undergoing surgical intervention for drug-resistant focal epilepsies. Given the wide-ranging diversity in the location, scale, and histological attributes of FCD, it's evident that a one-size-fits-all surgical approach would be inadequate. This underscores the imperativeness of devising and refining surgical techniques tailored to the unique challenges each FCD case presents.

However, even with these advances, certain challenges remain. One prominent area of ongoing research is the classification of FCD subtypes. Despite having multiple parameters at disposal, including histology, clinical data, electroencephalographic findings, and neuroimaging insights, achieving a consensus on a robust classification system is a work in progress [44]. Moreover, the task of detecting subtle FCDs, particularly when they manifest as normal on neuropathology or don't prominently feature in preoperative MRI scans, is a formidable diagnostic challenge. This highlights the pressing need to bolster our detection capabilities and fine-tune categorization methodologies.

Additionally, current research is diving into the realm of neuroinformatics to enhance the diagnosis and treatment of FCD. Advanced machine learning algorithms are being developed to analyze vast datasets of brain imaging and EEG recordings. The goal is to create predictive models that can accurately identify FCD lesions and forecast treatment outcomes, thereby opening a new frontier in FCD management [26].

Furthermore, the use of liquid biopsy techniques to identify biomarkers related to FCD is an evolving area of interest. These minimally invasive techniques could provide real-time data on the molecular changes occurring in the brain, offering a more dynamic understanding of FCD pathology [45].

The progress in gene-editing technologies, particularly clustered regularly interspaced short palindromic repeats (CRISPR), also holds immense promise. Current studies are exploring the use of CRISPR for targeted modification of genes associated with FCD, aiming for a more definitive cure rather than symptomatic management.

Another pivotal advancement in FCD research is the exploration of telemedicine and remote monitoring. With the ongoing global shift towards digital healthcare, telemedicine could play a crucial role in providing timely interventions and improving the quality of life for patients, especially in remote areas.

The integration of artificial intelligence in surgical planning represents a significant leap forward. Artificial intelligence (AI) algorithms can simulate various surgical approaches based on patient-specific variables, helping surgeons to choose the most effective and least invasive techniques. This is particularly relevant for FCD given its heterogeneous presentation, which often requires highly personalized surgical approaches.

Moreover, a promising avenue of exploration lies in the study of the mTOR pathway. Preliminary research hinting at its involvement in FCD offers tantalizing prospects for therapeutic interventions. Delving deeper into the intricacies of somatic mosaicism within the brain could potentially unlock groundbreaking treatment modalities and reshape our understanding of FCD's underpinnings [45]. As the field progresses, it's evident that a combination of refined diagnostic tools, personalized surgical techniques, and targeted therapies will be central to transforming the therapeutic landscape for FCD.

## Conclusions

FCD is a distinctive neurological condition characterized by localized abnormalities in brain cell organization, primarily affecting neurons. Particularly prevalent among children and young adults, FCD is closely tied to medically resistant epilepsy, making it a key focus in neurology. Its discovery and subsequent research have revolutionized our understanding of epilepsy and cortical abnormalities, informing both diagnosis and treatment. Clinical manifestations are diverse, including a range of seizure types, cognitive issues, and neuropsychiatric comorbidities. Diagnosis hinges on a multi-modal approach, employing intricate EEG interpretations, microscopic evaluations, genetic assessments, and a comprehensive understanding of clinical symptoms.

Cutting-edge tools like 3T MRI epilepsy protocols are invaluable for pinpointing FCD lesions. Treatment options range from anti-seizure medications to surgery for cases resistant to medical intervention. Surgical approaches include lesionectomy, lobectomy, and various disconnection strategies, all necessitating rigorous postoperative monitoring for optimal outcomes. The prognosis for FCD is a complex mix of fluctuating seizure control and cognitive and psychiatric sequelae, demanding personalized treatment plans. The field of FCD research is ripe with opportunities, from exploring new treatment options and surgical techniques to investigating the mTOR pathway. Each research and clinical milestone brings us closer to improving the lives of those grappling with FCD and its associated epileptic complications.
